# Selecting the correct cellular model for assessing of the biological response of collagen-based biomaterials

**DOI:** 10.1016/j.actbio.2017.10.035

**Published:** 2018-01

**Authors:** Natalia Davidenko, Samir Hamaia, Daniel V. Bax, Jean-Daniel Malcor, Carlos F. Schuster, Donald Gullberg, Richard W. Farndale, Serena M. Best, Ruth E. Cameron

**Affiliations:** a Department of Materials Science and Metallurgy, University of Cambridge, 27 Charles Babbage Road, Cambridge CB3 0FS, United Kingdom; b Department of Biochemistry, University of Cambridge, Downing Site, Cambridge CB2 1QW, United Kingdom; c Department of Biomedicine, University of Bergen, Jonas Lies vei 91, N-5009 Bergen, Norway

**Keywords:** Tissue engineering, Collagen, Cell adhesion, Integrins, Crosslinking

## Abstract

Accurate evaluation of the biological performance of biomaterials requires the correct assessment of their native-like cell ligation properties. However, cell attachment studies often overlook the details of the substrate-cell binding mechanisms, be they integrin-mediated or non-specific, and ignore the class- and species-specificities of the cell adhesion receptor involved. In this work we have used different collagen (Col) substrates (fibrillar collagens I, II and III and network-forming Col IV), containing different affinity cell-recognition motifs, to establish the influence of the receptor identity and species-specificity on collagen-cell interactive properties. Receptor expression was varied by using cells of different origin, or transfecting collagen-binding integrins into integrin-null cells. These include mouse C2C12 myoblasts transfected with human α1, α2, α10 or α11; human fibrosarcoma HT1080 cells which constitutively express only human α2β1, and rat glioma Rugli cells, with only rat α1β1. Using these lines, the nature of integrin binding sites was studied in order to delineate the bioactivity of different collagen substrates. Integrin ligation was studied on collagen coatings alongside synthetic (GFOGER/GLOGEN) and Toolkit (Col II-28/Col III-7) triple-helical peptides to evaluate (1) their affinity towards different integrins and (2) to confirm the activity of the inserted integrin in the transfected cells. Thin films of dermal and tendon Col I were used to evaluate the influence of the carbodiimide (EDC)-based treatment on the cellular response on Col of different origin. The results showed that the binding properties of transfected C2C12 cells to collagens depend on the identity of inserted integrin. Similar ligation characteristics were observed using α1+ and α10+ cells, but these were distinct from the similar binding features of α2+ and α11+ cells. Recombinant human and rat-α1 I domain binding to collagens and peptides correlated with the cell adhesion results, showing receptor class- and species-specificities. The understanding of the physiologically relevant cell anchorage characteristics of bio-constructs may assist in the selection of (1) the optimum collagen source for cellular supports and (2) the correct cellular model for their biological assessment. This, in turn, may allow reliable prediction of the biological performance of bio-scaffolds *in vivo* for specific TE applications.

**Statement of Significance:**

Integrins play a vital role in cellular responses to environmental cues during early-stage cell-substrate interaction. We describe physiologically relevant cell anchorage to collagen substrates that present different affinity cell-recognition motifs, to provide experimental tools to assist in understanding integrin binding. Using different cell types and recombinant integrin α1-I-domains, we found that cellular response was highly dependent on collagen type, origin and EDC-crosslinking status, as well as on the integrin class and species of origin. This comprehensive study establishes selectivity amongst the four collagen-binding integrins and species-specific properties that together may influence choice of cell type and receptor in different experimental settings. This work offers key guidance in selecting of the correct cellular model for the biological testing of collagen-based biomaterials.

## Graphical abstract

**Figure:**
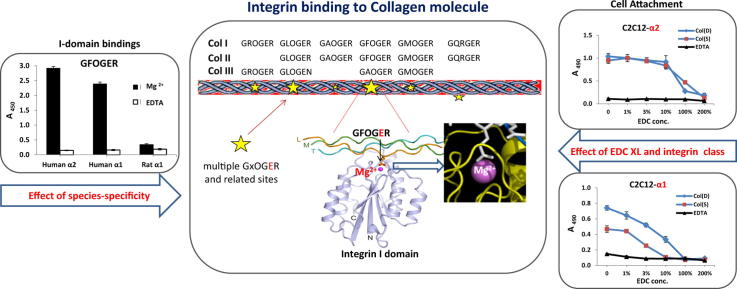

## 1 Introduction

Natural extracellular matrix (ECM) contains a mixture of proteins and polysaccharides that display biochemical cues which influence cell behaviour. This composition determines the cell-binding affinity through specific interaction with integrins presented on the cell surface [1], [2]. ECM components possess different adhesive motifs with diverse affinities towards a variety of cell recognition receptors. Despite this complex tissue composition, for many years, collagen (in forms including gels, scaffolds and membranes) has been a commonly used biomaterial due to its biocompatibility, biodegradability and low immunogenicity, together with its ability to form fibres with high tensile strength [2], [3], [4], [5]. Collagen (Col), being the principal structural protein in all vertebrates, comprises a family of genetically distinct molecules with a common triple helix configuration of three polypeptide subunits known as α-chains [4], [6]. These triple helices comprise a molecule of tropocollagen, the basic building block of collagen fibres. Tropocollagen molecules associate in a staggered fashion to produce collagen fibrils, which are strengthened and stabilized mainly by enzymatic and non-enzymatically catalysed covalent cross-links. The extent of these crosslinks is age-dependent and tissue-specific. The human genome contains 28 collagens and the corresponding proteins are made up of about 40 gene products, identified and described in varying detail [6]. Variations in collagen types are due to differences in the primary sequence and assembly of the polypeptide subunits, the lengths of the helix and the interruptions and terminations of the collagenous helical domains. The best known and the most abundant collagens are fibrillar collagens I, II and III, each containing different affinity cell-recognition motifs that support cellular activity mainly through their interaction with cell-associated integrins α1β1, α2β1, α10β1 and α11β1 [6], [7]. Col I is a major ECM component and accomplishes both structural and cell adhesive roles in many vital organs and tissues [3], [8]. Col II is the chief element in articular cartilage (approximately 60% of the dry weight of this tissue) [2], [9] while Col III is an important component of reticular fibres, where it is commonly found alongside Col I [10], for example in skin and blood vessel walls. These collagens have been used, alone or in combination, for the design of bio scaffolds [2], [3], [5]. Col I is the most widely-explored option, owing to its physical and biological attributes, the ability to isolate it to high purity and its reasonable cost. Despite this, the addition of other collagens may be highly beneficial. For example, the introduction of Col III seems advantageous when engineering cellular supports for cardiac tissue replacement as this collagen, in native tissue, plays an important role by linking contractile elements of adjacent myocytes [10]. The structural diversity observed in different Col types affects their adhesive motifs which may in turn have impact on their cell-substrate interactions via integrins [6], [7].

Integrins are transmembrane glycoproteins that represent a family of 24 heterodimeric signalling receptors each composed of a single α- and β-subunit. These play a central role in mediating dynamic cell–cell and cell–extracellular matrix/substrate interactions. Integrins recognise a large number of similar motifs presented in the different types of collagens. They are unique, among adhesion molecules, as their adhesiveness is dynamically regulated through “inside-out signalling,” which in turn leads to ligand binding and signal transduction in the classical “outside-in” direction [11], [12], [13], [14]. The strength of cellular adhesiveness of an integrin is largely governed by the intrinsic affinity of the individual receptor–ligand interface, which is dynamically modulated by conformational changes. Of the four collagen-binding integrins, α1β1 and α2β1 have been studied for almost three decades whilst the properties of both α10β1 and α11β1 are still not fully explored [7].

**Fig. 1:**
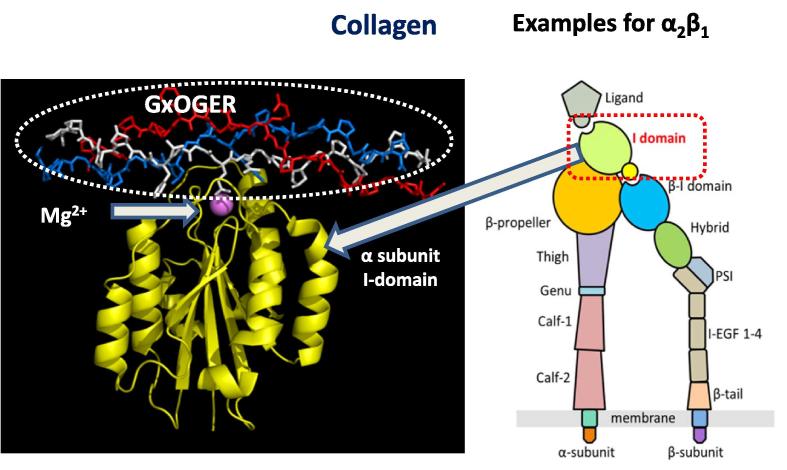
The crystal structures of integrin-α2 I domain and its Mg^2+-^dependent binding to GFOGER collagen motif was produced from pdb:1DZI. Schematic representation of α2β1 integrin with inserted I domain in the α subunit.

Adapted from [13]

All collagen-binding integrins are distinguished by the presence, within the α-subunit, of an inserted A-domain, termed an I domain. The I domain co-ordinates a divalent cation, Mg^2+^, in its metal ion dependent adhesion site, which is the principal site of interaction with collagens [7], [13], [15]. The crystal structure of integrin α2 I domain when interacting with Col triple-helical GFOGER motif has been resolved [15]. Fig. 1 schematically represents the position of this I domain in the α-subunit of α2β1 integrin and illustrates the complex formation between a collagen triple helical motif (GFOGER) and an integrin I domain via coordination with Mg^2+^. The mechanism of this interaction dictates that the carboxylate sidechain of the glutamic acid (E) residues in GxOGEx’ and the presence in the culture medium of the divalent cation (Mg^2+^) are critical for integrin-mediated binding. The removal of Mg^2+^ cations by chelation with ethylenediaminetetraacetic acid, EDTA, effectively blocks this mode of interaction and is frequently used as a convenient method to deconvolute this native-like adhesion from non-integrin-based cell attachment [16], [17].

As integrins α_1_β_1_ and α_2_β_1_ bind to collagen via these inserted I domains within the α-subunit, recombinant I domains offer the possibility to test for direct integrin-collagen engagement in a cell-free system, that is without interference/input of other cell-surface receptors and independently on the receptor density on the cell surface. As such this analysis can shed light on possible class-and species-specificities of integrin-substrate interactions.

Only the correct stimulation of cell-attachment mechanisms, through an intimate, biologically relevant cell-substrate ligation, leads to the further cell activity such as spreading and proliferation. Despite this, many cell attachment studies overlook the detail of the binding mechanisms, be they integrin-mediated or non-specific. Moreover, the class-and species-specificities of the cell adhesion receptor involved in the direct substrate-integrin association are frequently ignored. In this study, the binding abilities of all four of the collagen-specific integrins have been probed by means of cells with diverse species-specificity (murine, human and rat) and which express different collagen-binding receptors: (1) C2C12 mouse myoblasts stably transfected with human α1+, α2+, α10+ or α11+ β1 integrins; (2) HT1080 human fibrosarcoma, expressing human α2β1, and (3) Rugli cells derived from a rat glioma that express rat α1β1. The original C2C12, non-transfected mother cells, which do not express collagen-binding receptors, were used as a negative control. For this study, adhesion assays were carried out on monolayer coatings thereby removing any possible impact of the substrate physical material properties [18].

To verify the activity of the transfected cells, studies of collagen-binding were carried out alongside investigation of binding to synthetic peptides containing cell-affinity motifs GFOGER and GLOGEN, and on their Toolkit equivalents II-28 and III-7, respectively, which were obtained from libraries of overlapping triple-helical peptides. Both Toolkit samples are composed of an active guest sequence, encompassing the natural sequence of collagens II and III (Toolkit-II and III respectively) [7], [19], [20], [21], [22], [23]. A triple-helical peptide containing the sequence GFOGER (present in Col I, Col II and Col IV) is the highest affinity ligand for integrin α2β1, whilst GLOGEN (an important ligand in Col III) is the best cell-recognition motif for integrin α1β1. The Toolkit peptides II-28 and III-7 specifically possess GFOGER and GLOGEN motifs, respectively, in their 29-residue section of primary collagen sequence, and as such, were selected for cell attachment tests, taking into account that screening of Toolkit II and III on collagen-binding integrins has previously been effectively used in our lab for the identification of cell-binding sites within collagen [7], [23].

The influence of Col I source on its cell adhesive properties was studied on both monolayers and 2D films produced from insoluble bovine dermal and tendon-derived samples. Thin films were crosslinked (XL) with different dose of EDC (1-ethyl-3-(3-dimethylaminopropyl-carbodiimide hydrochloride) in the presence of NHS (N-hydroxy-succinimide), to evaluate the influence of the treatment conditions on the cellular response of different collagen substrates. Carbodiimide-based crosslinking constitutes one of the most successful chemical methods to modulate the mechanical stability and the dissolution resistance of collagenous materials. It is highly efficient, nontoxic, and by-products can be easily eluted [17], [24], [25], [26], [27], [28]. However, this procedure results in the consumption of carboxylate sidechains on glutamate (E) or aspartate (D) residues through their reactions with an adjacent lysine (K) ε-amino group [24], [29], [30]. Both glutamate and aspartate are important amino acid components of numerous cell-recognition ligands in collagen molecules and their depletion, as shown in [7], [17], [31], [32], may lead to the loss of integrin-substrate ligation.

Understanding the effect of the source and the type of collagen, before and after crosslinking treatment, on the biological behavior of collagenous materials may assist in the selection of the optimum components for the design of specific ECM-like devices to more closely match the properties of the tissues they aim to repair or replace. This study used cell lines that express single collagen-binding integrins as a tool to probe collagen ligation properties. The results show the importance of the receptor class- and species-specificities for cell-substrate interaction. Moreover, the work highlights the differential cell-recognition of different collagen types which is modified with ranging crosslinking densities and the importance of selecting the correct cellular model to assess the biological properties of protein-based biomaterials.

## 2 Materials and method

### 2.1 Materials

#### 2.1.1 Cell lines

HT1080 (fibroblasts from human sarcoma) cells were obtained from the European Collection of Animal Cell Cultures, Porton Down, UK. The parent C2C12 mouse myoblast cells and C2C12 cells, stably transfected with the human integrin α1, α2 and α11 subunits, were produced as described in [33]. The C2C12 cells stably transfected with integrin α10 were a kind gift from Dr. E Lungren-Akerland, Xintela AB, Lund, Sweden. Rugli (derived from a rat glioma) cells were a kind gift from Dr. J. Gavrilovic, University of East Anglia, Norwich, UK.

The human I domains from T141 to E335 for α1 and from S141 to E336 for α2 were expressed as recombinant N-terminal GST fusion proteins as detailed in [20], [34]. In the same way, rat α1 I domain has been cloned from Rugli cells fused to GST, started from S226 to E421.

#### 2.1.2 Materials

Insoluble microfibrillar collagens type I derived (1) from bovine Achilles tendon, termed Col I(S), was purchased from Sigma–Aldrich Co. Ltd. UK, and (2) from bovine epidermis layer in skin, termed Col I(D), was purchased from Devro Medical Bathurst, NSW, Australia. Soluble fibrillar collagens type II (Col II), from bovine tracheal cartilage, type III (Col III), from human placenta, and network soluble Col IV, also extracted from human placenta, were all purchased from Sigma-Aldrich Co. Ltd. UK. 9-Fluorenylmethoxycarbonyl (Fmoc) protected amino acids and N,N-Dimethylformamide (DMF), used for peptide synthesis, were supplied by AGTC Bioproducts (Hessle, UK). All other amino acids and reagents were purchased from Sigma-Aldrich (Gillingham, UK). Acetic acid (2 M), 1-ethyl-3-(3-dimethylaminopropyl)-carbodiimide hydrochloride (EDC) and N-hydroxy-succinimide (NHS) were purchased from Sigma-Aldrich Co. Ltd. UK. Dulbecco Modified Eagles Medium (DMEM), phosphate buffered saline (PBS), Foetal Calf Serum (FCS), penicillin and streptomycin were purchased from Invitrogen Life Sciences (UK). Other commercially available reagents were all analytical grade.

#### 2.1.3 Synthesis of GFOGER and GLOGEN peptides

Peptides were synthesized using method described in [22], [35]. Briefly, we used an Fmoc/*tert*-butyl solid phase strategy on a Liberty Blue™ microwave peptide synthesizer (CEM) on a 0.1 mmol scale, using HCTU as coupling reagent and DIEA as base. Peptides were assembled on Fmoc-Rink amide aminomethyl Tantagel resin (0.526 g, loading 0.19 mmol.g^−1^, RAPP Polymere) to yield C-terminal amides. Fmoc removal was performed with piperidine in DMF (20% v/v). Successive amino acid couplings and deprotection steps were carried out in DMF under microwave radiation. After peptide assembly, resin beads were washed with dichloromethane (DCM) twice, methanol (MeOH) twice and DCM. Cleavage from the resin was performed over 2 h in 10 ml of a mixture of trifluoroacetic acid (TFA)/triisopropylsilane (TIS)/H_2_O 95/2.5/2.5 v/v/v with 250 mg of dithiothreitol (DTT). The cleavage solution was concentrated and precipitated in 20 ml of cold diethyl ether. The white precipitate was filtered, washed with 10 ml of cold diethyl ether twice and redissolved in a H_2_O/acetonitrile (ACN) 95/5 v/v (0.1% TFA) mixture. The crude product was recovered after freeze-drying and purified by preparative reverse-phase high performance liquid chromatography (RP-HPLC) on a Perkin Elmer LC200 system equipped with a 10 μm Eurospher II 100-10 C18 H (Knauer, Berlin, Germany) with a linear gradient of ACN 0.1% TFA in water 0.1% TFA. Purified compounds were characterized by matrix-assisted laser desorption ionization time-of-flight (MALDI-TOF) mass spectrometry at the Protein and Nucleic Chemistry Facility (University of Cambridge, Cambridge, UK). Full sequence of GFOGER and GLOGEN peptides are GPC(GPP)_5_**GFOGER**(GPP)_5_GPC and GPC(GPP)_5_**GLOGEN**(GPP)_5_GPC, respectively. The control triple-helical collagen-like peptide GPP10 (complete sequence, GPC(GPP)_10_GPC) was synthesized as described previously [19], [22].

#### 2.1.4 Toolkit II-28 and III-7peptides

Toolkit peptides II-28 (GPC(GPP)_5_GEAGAOGLVGPRGER**GFOGER**GSOGAQ(GPP)_5_GPC) and III-7 (GPC(GPP)_5_GETGAOGLKGEN**GLOGEN**GAOGPMGPR(GPP)_5_GPC were produced using the method described in [22].

### 2.2 Tested substrates

#### 2.2.1 Monolayer coated surfaces

Collagens and peptides coatings were produced on the surface of Immulon 2HB 96-well plates (Thermo Scientific) by incubating 100 µl/well of 10 µg/ml solution or suspension (for insoluble collagen type I) in 10 mM acetic acid containing the appropriate proteins/peptides over night at 4 °C. Bovine serum albumin (BSA, Sigma) and triple-helical-like sequences GPP10 were plated in triplicate to act as nonspecific background adhesion controls.

#### 2.2.2 Films

First, slurries of insoluble collagen type I (derived from bovine tendon Col (S) or skin Col(D)) were produced by swelling at 0.5% (w/v) in 50 mM acetic acid at 4 °C overnight and then homogenising on ice for 30 min at 13500 rpm using an Ultra-Turrax VD125 (VWR International Ltd., UK). Air bubbles were removed from slurries by centrifuging at 2500 rpm for 5 min (Hermle Z300, Labortechnik, Germany). Collagen films of ∼8 µm thickness were prepared by pipetting 100 μL/well of these slurries directly in Immulon 2HB plates (Thermo Scientific) and drying for 48 h in a laminar flow cabinet.

Films were cross-linked (XL) with carbodiimide (EDC) in combination with succinimide (NHS). An EDC concentration of 11.5 mg/ml, with a molar ratio of EDC/NHS/COO^-^(Col) = 5/2/1 in 95% (v/v) ethanol, was taken as standard (100%) and was varied from 1 to 200%. These crosslinking conditions were selected on the basis of our previous work where the effect of the reducing of standard crosslinking concentration down to very low levels (up to 1% EDC) on different relevant material and some cell-interactive properties was elucidated [24], [32]. After reaction for 2 h at room temperature, the films were washed thoroughly in deionised water (15 min × 5) and dried in a laminar flow cabinet.

### 2.3 Cell adhesion and spreading

Cell adhesion in the presence of Mg^2+^ (integrin-mediated) and EDTA (non-divalent cation specific) was assessed by colorimetric assay using a cytotoxicity detection kit (LDH), Roche, Cat. No 11 644 793001. This assay is based on the measurement of lactate dehydrogenase (LDH) activity release from lysed cells into the media.

#### 2.3.1 Cells adhesion on surfaces and films

All cell lines were maintained in a humidified incubator with 5% CO_2_ at 37 °C in DMEM, containing 10% fetal bovine serum and 1% streptavidin/penicillin. Prior to cell adhesion experiments, cells were detached from the cell culture flasks with 0.05% trypsin/0.02% EDTA (GE Healthcare), washed and re-suspended in serum free DMEM.

Before cell addition, non-specific adsorption to the coatings/films was blocked with 200 μl of bovine serum albumin (BSA, 5% (w/v) in PBS) for 60 min, and then wells were washed three times with 200 μl of PBS. 100 μl of cell suspension at different concentrations (from 0.5 to 4 × 10^5^ cells/ml in serum free DMEM) containing either 5 mM MgCl_2_ or 5 mM EDTA, were added to wells and allowed to attach at room temperature for 60 min. The wells were thoroughly washed with PBS (200 μl ×3) to remove loosely bound cells and then 50 μl of lysis buffer containing 2% v/v Triton X-100 in distilled water was added for 90 min at room temperature. Subsequently, 50 μl of LDH detection substrate (cytotoxicity detection kit (LDH), Roche, Cat. No 11 644 793001), prepared according to the manufacturer’s instructions, was added and left until color had sufficiently developed as to be accurately detected (typically from 10 to 30 min depending on the tested system). The variation in time point influences the absolute absorbance value but does not affect the adhesion profiles. The absorbance was read at 490 nm (A_490_) using a Fluostar Optima plate reader (BMG Labtech). Background adhesion was determined on BSA and GPP10 coated plates. Cell adhesion assays were performed in triplicate and values are reported as means ± standard deviations.

For quantitative analysis calibration curves, OD (optical density) *vs* known cell concentration, were obtained for each experiment. These were constructed by taking 500 µl of cell suspension at a known cell density and subsequently diluting 32 to 64 times. Cells were separated by centrifuging, lysed by adding of 250 μl of buffer containing 2% v/v Triton X-100 in distilled water for 90 min at room temperature, vortexed and then 50 µl aliquots of each solution were pipetted by triplicate in the same well plate in which the corresponding cell attachment test was conducted. After that 50 μl of LDH detection substrate were added to the calibration series at the same time as to the coated wells and left until color had developed (from 10 to 30 min). The absorbance of this series was read under the same conditions/time as on coated wells. A linear regression was fitted to the known cell values, which was used to calculate the cell number in the experimental wells. The selection of the time point for absorbance reading (color development) has no impact on the percentage of adhesion calculated using calibration curves due to the same level of color development on calibration solutions and on tested wells.

#### 2.3.2 Cell spreading tests

For spreading analysis, 100 μl of cell suspension at 1 × 10^5^ cells/ml containing either 5 mM Mg^2+^ or 5 mM EDTA in serum free DMEM was added to BSA blocked surfaces for 90 min at 37 °C/5% CO_2_. The cells were fixed by the addition of 9 μl of 37% (w/v) formaldehyde (final concentration 3.7%) directly to the cell media for 20 min at room temperature. The samples were washed with 3 × 200 μl PBS then viewed using a LEICA DMI6000CS phase contrast microscope fitted with a LEICA DFC340FX camera. Cell spreading (percentage of spread cells versus total number of cells) was determined by analyzing 12 images per condition. A cell was scored as spread if it was phase-dark with cellular projections and a flattened morphology. Cells were scored as non-spread if rounded and phase-bright with no cellular projections as detailed in [18], [36]. The percentage cell spreading was calculated by dividing the number of spread cells by the total number of cells present. Values are means of triplicate or quadruplicate measurements ± standard deviation.

### 2.4 Integrin I domain binding analysis

Prior to I domain binding, coated plates were blocked with 5% (w/v) BSA in binding buffer tris-buffered saline (TBS) (50 mM TRIS, 140 mM NaCl, pH 7.4, containing 1 mg/mL BSA) for 1 h at room temperature. Following BSA blocking, the samples were washed with 3 × 200 μL of binding buffer then incubated in 10 μg/mL of the corresponding recombinant integrin α1 (human or rat) or α2 (human) I domain in binding buffer containing either 5 mM MgCl_2_ or 5 mM EDTA. After 1 h incubation at room temperature, the samples were washed in 3 × 200 μL of binding buffer containing 5 mM MgCl_2_ or 5 mM EDTA respectively. The presence of the GST tagged recombinant I domain was detected by incubating with 100 μL of 1:10,000 diluted HRP conjugate, Amershan GE Healthcare RPN1236, for 1 h at room temperature. The detection antibody was removed and the films were washed with 5 × 200 μL of binding buffer for each wash. 100 μL of TMB substrate (Thermo Scientific) was added to each well and the reaction was stopped by the addition of 100 μL of 2.5 M H_2_SO_4_ to each well. A_450_ was measured using a Spectra Max 190 (Molecular Devices). Values are means of triplicate or quadruplicate measurements ± standard deviation.

### 2.5 Statistical analysis

Unless otherwise stated all error bars indicate standard deviations. Statistical significance was determined with a student *t*-test with unequal variance where N/S indicates none statistically significant (p > .05), ^∗^indicates p ≤ .05, ^∗∗^indicates p ≤ 0.01, ^∗∗∗^indicates p ≤ .001 and ^∗∗∗∗^indicates p ≤ 0.0001. All statistical annotations in films indicate the statistical difference between the data point annotated and the 0% crosslinking values.

## 3 Results

### 3.1 Cell adhesion and spreading on collagen coatings and peptides

Cell adhesion on coatings was carried out alongside synthetic (GFOGER and GLOGEN) and Toolkit, II-28 and III-7, peptides (1) to evaluate their affinity towards different integrins and (2) to confirm the activity of transfected cells. Short synthetic peptides possess one cell recognition ligand (GFOGER or GLOGEN) flanked by five GPP sequences and one GPC triplet on both the N and C terminal sides, which promotes a triple-helical configuration and ensures their attachment to the plastic well surface. The selection of these peptides was based on literature reports showing that Toolkit II-28 peptide and its derivative GFOGER are high affinity ligands for integrins α2β1 and α11β1 [19], [37] while peptides III-7 and the corresponding GLOGEN were good ligands for α1β1 and α10β1 [20], [22]. An inactive peptide containing only the flanking sequences GPP10 (GPC(GPP)_10_GPC) was used as a negative control in this study.

**Fig. 2:**
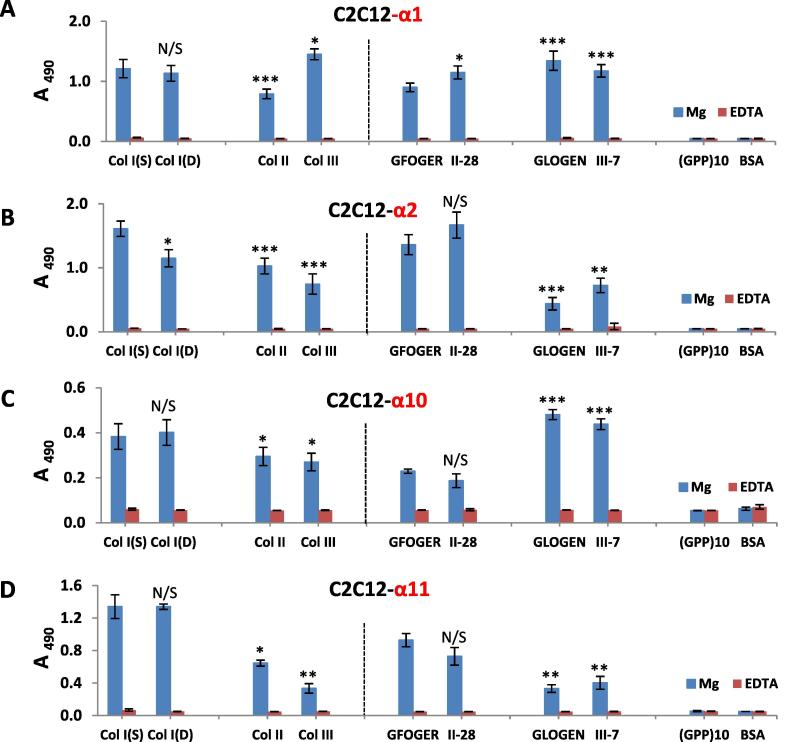
A representative data set showing the adhesion (optical density at 490 nm) profiles on Col coatings and peptides (n = 3 for each condition). Cell concentration 1.5 × 10^5^ cells/ml A–D adhesion of C2C12 cell line transfected with human α1+, -α2+, -α10+ and α11+ integrins, respectively. Statistical significance is shown compared to Col I(S) for all collagen types and to GFOGER for all peptides.

Magnesium-dependent and non-specific, cation-independent (EDTA) adhesion profiles for C2C12 cell line transfected with human α1+, -α2+, -α10+ or α11+ integrins on different Col coatings and peptides are presented in Fig. 2. It can be observed that all adhesion is Mg^2+^-dependent, and so integrin-mediated for all integrin transfected C2C12 cells. Original non-transfected C2C12 parent cells, which do not endogenously express collagen-recognition receptors, do not adhere to any substrate (data not shown). The ligation properties to Col I (S, D) for α2 and α11–positive C2C12 follow the affinity Col I > Col II > Col III (Fig. 2B and D). For C2C12-α1+ cells (Fig. 2A) there is a significant increase in adhesion on Col III in comparison with Col I and especially Col II. The level of adhesion of α10+ cells on Col II and Col III was similar but lower than on Col I (Fig. 2C).

The adhesion profiles on synthetic triple-helical (GFOGER and GLOGEN) and Toolkit peptides followed the published trends [7] thereby confirming that all transfected C2C12 cells correctly express the cloned integrin. Furthermore, very similar adhesion levels were observed on the short GFOGER and GLOGEN and their longer corresponding Toolkit peptides for all transfected cells. The binding properties of C2C12 with α1+ and –α10+ integrins appear most similar (Fig. 2A and C). This was distinct from the comparable ligation characteristics of α2+ and -α11+cells (Fig. 2B and D). The adhesion values on GFOGER and II-28 peptides were similar to those observed on full length Col I molecules for C2C12-α1+, -α2+ and α11+ transfected cells. C2C12-α1+ and -α10+ positive cells showed the highest attachment properties to GLOGEN and III-7 peptides which were comparable to the level of adhesion found on Col III, especially in the case of α1+ cells.

**Fig. 3:**
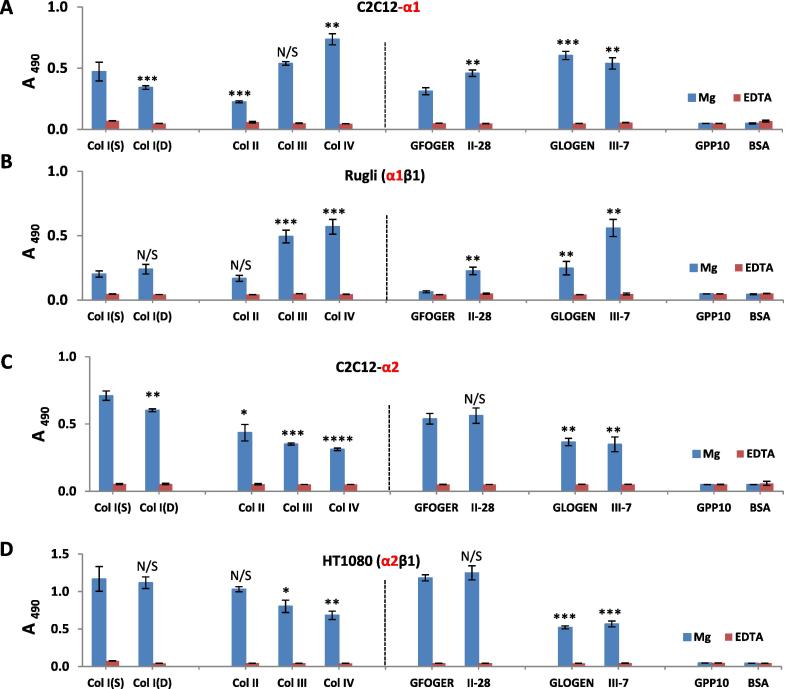
Representative profiles of C2C12-α1+ (A), Rugli (B), C2C12-α2+ (C) and HT1080 (D) cells at cell count 1 × 10^5^ cells/ml. Statistical significance is shown compared to Col I(S) for all collagen types and to GFOGER for all peptides.

To test the activity of endogenously expressed rather than transfected integrins, cell adhesion assays were also carried out using model cell lines HT1080 (human fibrosarcoma) and Rugli (rat glioma). These each express a single, specific collagen-binding integrin; as α2β1 and α1β1, respectively. Fig. 3 shows typical adhesion profiles for Rugli and HT1080 cells (Fig. 3B and D) together with those found for C2C12-α1+ and C2C12-α2+ cell lines at the same cell count (Fig. 3A and C). Col IV was included as it is a known specific marker for cells expressing α1β1 integrin.

Comparison of adhesion results for C2C12-α1+ and Rugli, α1β1, (Fig. 3A and B) showed that although both cell lines (with human and rat α1β1 integrin, respectively) exhibited similar adhesion trends (Col IV > Col III > Col I > Col II), the level of adhesion on Col I and, especially on GFOGER, was significantly lower for Rugli than for C2C12-α1+. This may be due to different expression levels or could suggest differences in affinities of human and rat α1 receptors to different triple-helical peptides present on Col I. For the human cell line expressing α2β1 integrin (HT1080) and mouse myoblasts C2C12 with human -α2+, the adhesion patterns were the same: Col I > Col II > Col III > Col IV.

Cell adhesion studies were carried out for different cell seeding densities to assess the possible influence of this parameter on adhesion profiles. It was found that for all transfected C2C12 cells (with all Col-binding integrins) the level of cell binding was greater on Col I(S) than on Col I(D) at lower cell counts (below 1.5 × 10^5^ cells/ml). This effect can be seen in Fig. 3A and C for C2C12-α1 and α2 positive cells where adhesion values on Col I(S) are higher than on Col I(D) for cell count 1 × 10^5^ cells/ml. At cell densities higher than 1.5–2 × 10^5^ cells/ml, similar adhesion values were observed on both dermal and tendon collagens. HT1080 and Rugli cells were not sensitive to the collagen I source as the level of attachment was the same on Col I(S) and Col I(D) at any cell density studied (examples are given in Fig. 3B and D).

**Fig. 4:**
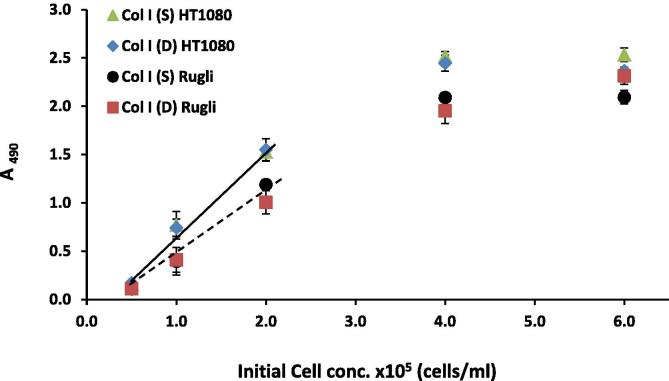
Examples of adhesion dependence on initial cell concentration for Col I samples.

Adhesion dependence on the cell seeding density showed that Mg^2+^-dependent adhesion linearly increased with the initial cell concentration in a range from 0.5 to 2 × 10^5^ cells/ml but at higher values this linearity was lost reaching saturation at cell densities above 3-4 × 10^5^ cells/ml (Fig. 4). Adhesion percentages calculated from calibration curves using a cell density within the detection range are presented in Table 1. These results show that the adhesion percentage on Col I (S and D) coatings is higher for cells expressing integrins α2 (HT1080 and C2C2-α2+) than for cells with integrin α1. Col III and Col IV samples were more adhesive for cells with α1 integrin in comparison with α2 receptor. This data also highlight the differences in ligation properties between human α1 and rat α1 integrins: for example the adhesion percentages of C2C12-α1+cells on Col I (S and D) were noticeably higher than for Rugli cells. On the other hand, the absolute values of adhesion percentages (showed in Table 1) seem quite low which may be a consequence of the experimental conditions chosen for the cell adhesion assays: at room temperature, for relatively short durations, without CO_2_ supply, and using a stringent washing regime to eliminate all loosely-bound cells. The level of adhesion will also reflect the level of receptor expression, not quantified here. As a result, these adhesion levels represent physiologically relevant cation-dependent integrin-mediated anchorage of the selected cell lines to the collagen substrates. This level of adhesion agrees well with our previous reports, e.g. [18].

**Table 1:** Percentage of cell adhesion expressing Col-binding integrins on different Col surfaces.

Col Type	HT1080 (α2β1)	C2C12-α2	Rugli (α1β1)	C2C12-α1
*Adhesion (%) for cell count 1 × 10^5^ cells/ml*				
Col I (S)	45.6 ± 6.4	44.2 ± 2.2	22.8 ± 2.7	36.4 ± 2.9
Col I (D)	43.6 ± 3.0	36.5 ± 0.5	27.0 ± 4.1	34.8 ± 2.7
Col II	40.3 ± 1.3	23.9 ± 3.0	18.9 ± 2.6	23.4 ± 1.5
Col III	28.1 ± 1.6	18.5 ± 0.5	55.6 ± 5.5	44.2 ± 2.5
Col IV	24.7 ± 2.0	16.1 ± 0.5	64.1 ± 6.4	51.9 ± 3.2

**Fig. 5:**
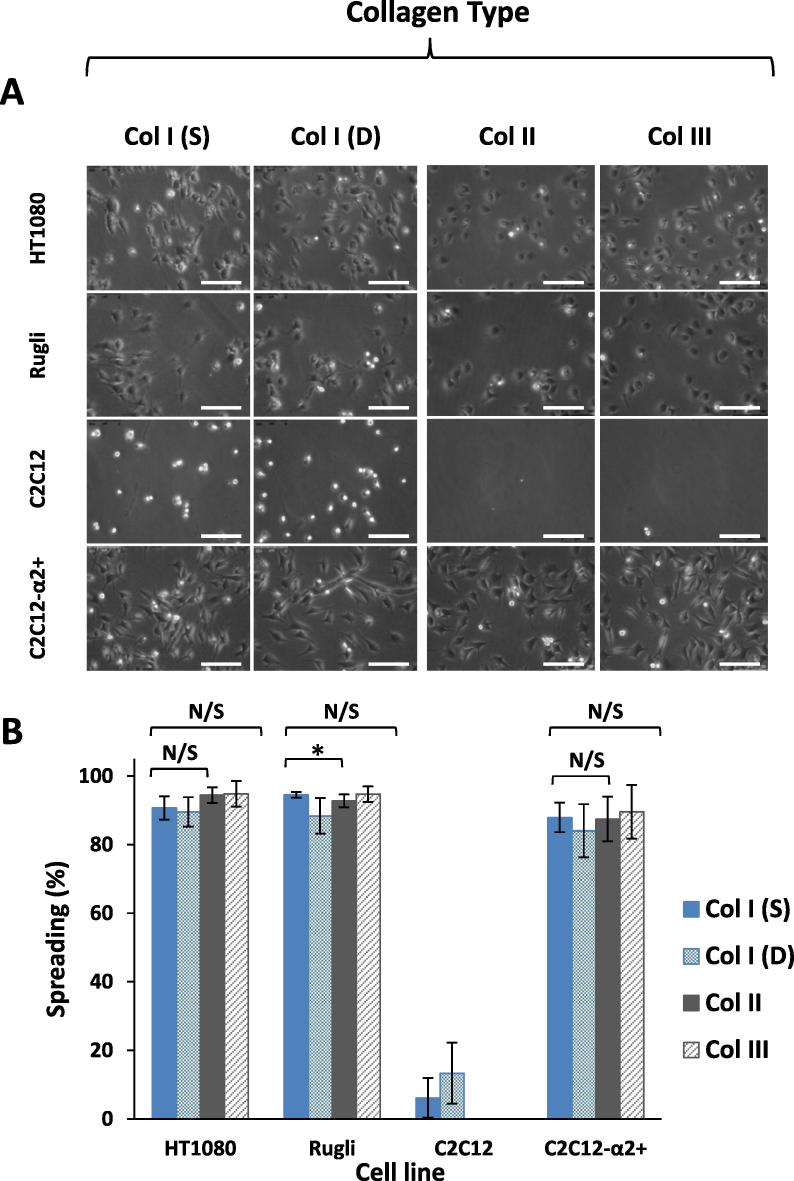
Images of cell spreading (A) of HT1080, Rugli, C2C12 and C2C12-α2+ cells and spreading percentage (B) on collagen surfaces. Scale bar is 100 µm.

Cellular spreading assays were performed on fibrillar collagen coatings using HT1080, Rugli and C2C12-α2 positive cells. C2C12 original non-transfected mother cells were employed as a negative control. The aim of this study was to determine whether the cell adhesion noted earlier leads to cell spreading as a result of the correct stimulation of certain signaling pathways after attachment. These assays were conducted in the presence of Mg^2+^ as no cell spreading was observed in the presence of EDTA (data not shown). It should be mentioned that these tests were carried out in the absence of serum in the cell media to prevent cell adhesion to serum containing proteins such as vitronectin and fibronectin that can bridge between Col and cells. Images showing the cell shape in the presence of Mg^2+^ for HT1080, Rugli, transfected C2C12-α2+ and “blank” C2C12 are displayed in Fig. 5A. It can be observed that all cell lines with collagen-binding integrins (HT1080, Rugli and C2C12-α2+) are similarly spread on all Col coatings: almost all attached cells look flattened with a phase dark appearance. Spreading quantification (Fig. 5B) showed a high degree of cell spreading (between 95 and 85%) for all Col-based surfaces. Non-transfected C2C12 cells were not spread on any collagen samples; in the case of Col I coatings, where some attachment was observed, all cells were phase bright and rounded.

### 3.2 Cell adhesion on Col I films

**Fig. 6:**
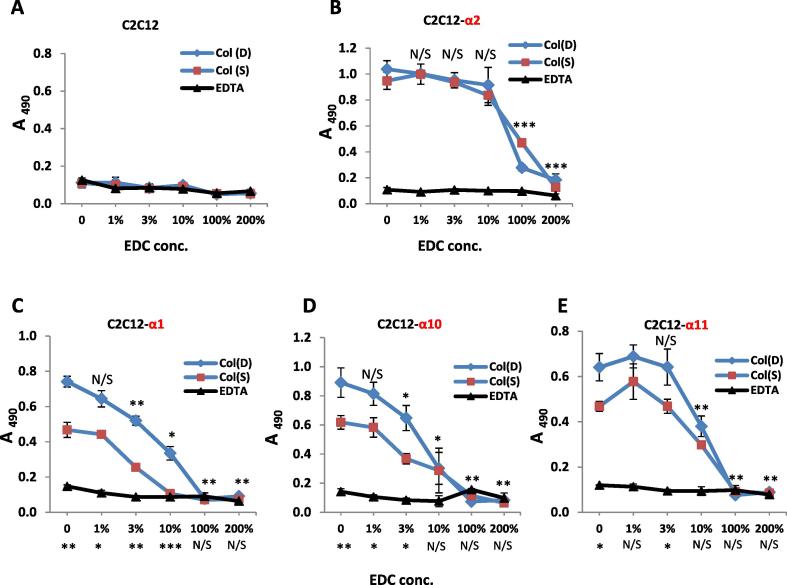
Adhesion profiles of C2C12 cells (A), C2C12 transfected with human -α2+(B), α1+(C),-α10+(D) and -α11+(E) integrins on Col-I films with different crosslinked status. Initial cell concentration: 3 × 10^5^ cells/ml. Statistical significances compared to 0% XL Col samples are shown above the data points. Statistical differences between Col I(S) and Col I(D) for the same crosslinking condition are shown below the “X” axis. For C2C12 and C2C12-α2 cells there are no significant differences between the adhesion values on Col I(S) and Col I(D) films with the same crosslinking conditions.

Fibrillar collagen I samples used in films and in coatings were analyzed for their amino acid content (as described in our previous works [17], [29]). Equivalent total protein content was observed in the as-received collagen preparations of insoluble Sigma and Devro collagen with 733 ± 13.1 μg/ml (S.D. n = 3) and 737.2 ± 35.5 μg/ml (S.D. n = 4), respectively. Films produced from these collagens were EDC cross-linked to establish the effect of collagen origin and the influence of variations in crosslinking conditions on cell behaviour on 2D samples. The same method for film preparation was used for both Col I samples. It was developed, validated (in term of collagen alignment, the surface structure, covering quality, thickness, etc.) and reported in our previous works [29]. For example, it was showed that the employed method provided films with the aligned fibre bundles of collagen within the plane of the films, homogeneously covering the underlying surface with similar topographic characteristics and thickness (in the range of 7–10 µm) for both non-treated and crosslinked samples. It was also demonstrated that EDC crosslinking did not induce any non-uniform changes in film morphology or protein distribution. Fig. 6 shows the adhesion profiles of C2C12 non-transfected mother cells (Fig. 6A) and C2C12 myoblasts transfected with all collagen-recognition integrins (Fig. 6B–E) on Col(S) and Col(D) films crosslinked with different concentration of EDC (from 0 to 200%). No adhesion of C2C12 “blank” cells was detected on any film. This was expected as this cell line does not express any collagen-binding integrin (Fig. 6A). All adhesion of transfected C2C12 cells was Mg^2+^-dependent (Fig. 6B–E) and the profiles share similarity in their dependence on EDC crosslinking dose: adhesion decreases with increasing EDC crosslinking. It can be observed that collagen origin influences the degree of adhesion, which, in addition, was sensitive to the integrin class involved in cell-substrate ligation. For example C2C12-α2 cells adhered similarly to both dermal and tendon Col samples (Fig. 6B) at all crosslinking concentrations. Conversely, for the other transfected cells (C2C12-α1, -α10 and -α11+) Mg^2+^-dependent adhesion was higher on dermal, Col (D), in comparison with tendon, Col(S), at all XL conditions except 100 and 200% where no cell binding was observed (Fig. 6C–E). Adhesion patterns were similar for -α2+ and α11+positive cells: collagen ligation was not affected with up to 10% EDC crosslinking (Fig. 6B and E). By contrast α1+ and α10+ C2C12 cell attachment showed dose-dependent inhibition even with very low EDC concentrations (1% and 3% for Col (D) and Col (S), respectively, Fig. 6C and D).

**Fig. 7:**
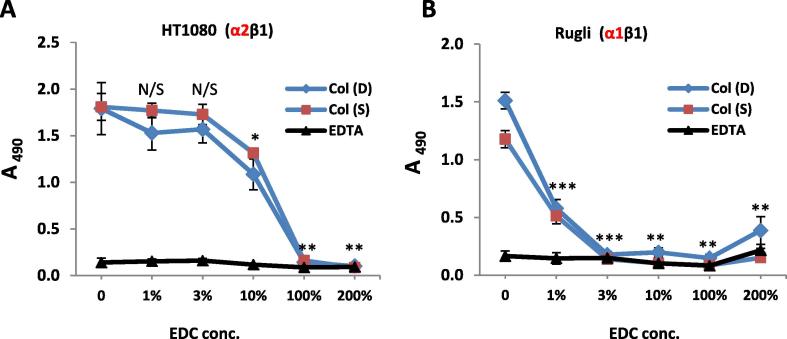
Adhesion profile of HT1080 (A) and Rugli (B) cells on Col-I films with different crosslinked status. Initial cell concentration: 3 × 10^5^ cells/ml. Statistical significance compared to 0% XL Col I(S) is shown above each data point. There are no significant differences between the adhesion values on Col I(S) and Col I(D) films with the same crosslinking conditions.

The adhesion of human fibroblasts HT1080 (α2β1) and rat glioma Rugli cells (α1β1) to Col I films with different crosslinking states was also tested (Fig. 7). For each cell line, the adhesion profiles were not dependent on Col origin as adhesion values on Col(S) and Col((D) were very similar for all crosslinking conditions used. However, the sensitivity of HT1080 and Rugli attachment to EDC crosslinking was very different. HT1080 (α2β1) attachment started to decrease from 10% EDC concentration onwards, showing the same profiles to those found for C2C12-α2+ (Fig. 6B). However, for Rugli cells (rat α1β1) a dramatic drop in adhesion values was observed on both dermal and tendon Col films after crosslinking treatment with only 1% of EDC (Fig. 7B). This pattern is significantly different to that observed for C2C12-α1+ cells (Fig. 6C) suggesting different response of cells transfected with human compared to endogenous rat α1-integrins.

### 3.3 Attachment of -α1 human I-domain and -α1-rat I domain on different collagens and selected synthetic and Toolkit peptides

**Fig. 8:**
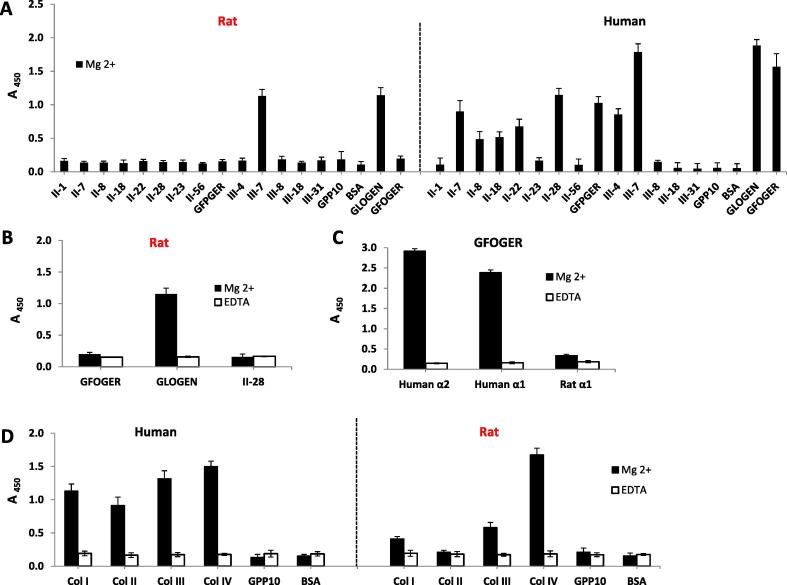
Attachment of α1 I domain (human and rat) on different collagens and selected synthetic and Toolkit peptides: A – rat and human α1 I-domains binding to selected Toolkit and synthetic peptides. EDTA controls block all binding to peptides (data not shown); B – α 1 I-domain binding to GFOGER, GLOGEN and II-28 in presence of Mg ^2+^ and EDTA; C – rat α1 vs human α1 and α2 I-domains binding to GFOGER; D – human vs rat α 1 I-domains binding to different collagens.

Attachment tests were carried out using purified recombinant I domains derived from α1-subunits of human and rat integrins. The aim of this study was to understand whether differences found in the interactive properties of cells expressing human α1- (C2C12-α1+) and rat α1- (Rugli) receptors with collagen substrates were due to (1) different expression levels on the corresponding cell surfaces or (2) due to differences in affinities of α1-human and α1-rat receptors towards collagen binding ligands. Additionally, this analysis could shed light on the different response of cells with human and rat α1 integrins to EDC crosslinked films. ELISA-based detection of I-domains bound to the selected synthetic and Toolkit peptides (Fig. 8A–C) and to different collagen types (Fig. 8D) showed significant differences in the adhesive properties between human and rat α1 I domains. Human integrin α1 I domain ligates to a greater variety of collagen derived triple-helical ligands, although GLOGEN sequences (GLOGEN peptide and Toolkit III-7) show the greater affinity. By contrast rat α1 I domain shows affinity only to the GLOGEN motif and to Toolkit III-7 which contain the GLOGEN ligand (Fig. 8A, B). Human α1 I domain ligation to GFOGER peptide and GFOGER-containing II-28 was very high and comparable with the level of adhesion found for the human α2 I domain. Human α2-GFOGER interactions are considered high affinity [7], therefore this human α2 control was included to allow direct comparison showing that human α1, but not rat α1, has a similar affinity to GFOGER as human α2 I domain (Fig. 8A and C). In contrast, almost no adhesion was detected for rat α1 I domain on GFOGER and on II-28 where the absorbance signal was near to the background level and to that in the presence of EDTA, Fig. 8A, B and C. Integrin α1 human I domain and rat α1 I domain show different affinities of ligation to different collagen types (Fig. 8D). The binding of human α1 I domain on Col I, II and III was considerably greater than that of the rat α1 I domain to the same collagen samples (Fig. 8D). However, the level of adhesion on Col IV was similar for both human and rat α1 I domains.

## 4 Discussion

### 4.1 Adhesion and spreading on coatings

Monolayer coatings allow the detection of integrin recognition sequences in collagen substrates by evaluating the affinity and availability of their biological cues towards variety of cellular receptors. These coatings were prepared from diluted (well below their saturation limits), highly homogenised solutions/suspensions of the corresponding collagen substrates. By using an appropriate coating concentration, this assessment is independent of the material bulk properties since only single molecule layers are deposited onto the solid cell culture plastic, so creating a surface-bound assembly of integrin recognition sequences typical to each collagen type. Three fibrillar collagens (types I, II and III) and the network Col IV, known as a selective ligand for α1β1 integrin [7], were chosen for this study to examine the cell-recognition sequences that support integrin ligation [6], [7]. As these collagens possess different solubility, the effect of this parameter on their surface immobilization was investigated. Enzyme linked immunosorbent assays (see supplementary data on collagen detection) show identical detection of collagen on coatings derived from insoluble Col I suspensions or a soluble Col I solution. This demonstrates that when incubated at excess, the solubility of the starting materials does not play an important role in the total surface coverage, which, in turn, provides the basis for comparing the response of cells expressing defined receptors to the different collagens. Through this analysis, the importance of the structural and sequence diversity on their interaction with integrin receptors was determined. In our previous work, we have shown that macromolecular triple-helical structures of Col I may display differences in their fiber architecture (fiber lengths, width, entanglement), when derived from different tissues (dermal and tendon). In turn, these structural modifications induce changes in the material properties of 3D scaffolds produced from these precursors [24]. Here, we extend upon this to determine whether these changes also impact on the biological function of this protein. C2C12 cells transfected independently with each of the four collagen-binding integrins showed that at low cell counts (within the linear concentration dependent zone) tendon collagen seems to be more cell-adhesive than its dermal equivalent. This may be a consequence of differences in the exposure of cell-adhesion sequences in these samples. Furthermore, as these collagen preparations possess different physical properties such as fibre diameter and stiffness, the differential cell adhesion could be due to surface-induced conformational and/or orientation changes, as observed previously for gelatin-coated surfaces [18].

The differential cell binding to collagen type I, II, III and IV may be accredited to the differences in the specific GxOGEx’ motifs and their corresponding affinities towards different Col-binding integrins. The distribution of GxOGEx’ sequences across fibrillar collagens have been previously identified (Table 2) [7] by using homotrimeric collagen-derived triple-helical peptide libraries called Toolkits. These assays also established that the affinity order in GxOGER ligands towards α2β1 integrin ligation showed the trend where was x = F > L ≥ R > M > A [7], indicating that GFOGER (found in Col I and Col II) is the highest affinity ligand for this receptor. Our results agree with relative affinities. We observed the highest adhesion values of C2C12-α2+ and HT1080 cells, both expressing α2β1 integrin, on Col I/Col II coatings and on GFOGER/Toolkit II-28 peptides (Fig. Fig. 2, Fig. 3C and D and Table 1). C2C12-α11+ myocytes showed very similar binding properties to α2-positive cells on collagen monolayers, which followed the trend Col I > Col II > Col III, and on collagen Toolkit peptides (Figs. Fig. 2, Fig. 3C and D). This suggests that GFOGER is a high affinity ligand for integrin α11. A high level of C2C12-α1+ and Rugli (α1β1) cell adhesion on Col III/IV and GLOGEN/Toolkit III-7 peptides is consistent with the presence of the GLOGEN motif within all of these substrates, as this motif has been identified as a high affinity sequence for integrin α1β1 [7], [22]. Col IV promoted high levels of adhesion for cells containing integrin α1 (Fig. 3A, B) which agrees with the definition of this network collagen as a selective ligand for α1β1 integrins [7]. It has also been reported that the affinity of GLOGEN for integrin α1β1 is comparable with that of GFOGER [7] which is in accordance with the high attachment levels to Col I and GFOGER/II-28 peptides we observed on C2C12 transfected with human integrin α1 (Figs. Fig. 2, Fig. 3A). Conversely Rugli cells, expressing rat integrin α1, showed very low adhesion levels on GFOGER which may explain the low attachment to Col I and Col II as these contain GFOGER as their primary cell-binding motif (Fig. 3B). Differences in the cellular response of Rugli and C2C12-α1+cells cannot be attributed to differences in the receptor density on the cell surface as both cell lines show very similar integrin-dependent adhesion to Col IV and on Toolkit III-7 peptides. Instead this points to different affinities between rat and human integrin α1 towards certain cell recognition sequences such as GFOGER. Therefore this suggests that not only integrin class, but also the species-specificity, may affect collagen-receptor interactions. Thus we highlight the importance of using humanised test systems for cell adhesion analysis.

**Table 2:** Integrin recognition sequences in fibrillar collagen (data from [7]).

Type of fibrillar collagen	Integrin recognition sequences	Single-letter amino acid code
Collagen I	GFOGER, GLOGER, GROGER, GMOGER	**G** Glycine **P** Proline **A** Alanine **L** Leucine **M** Methionine **F** Phenylalanine **R** Arginine **N** Asparagine **E** Glutamic Acid **D** Aspartic Acid
Collagen II	GFOGER, GLOGER, GMOGER	
Collagen III	GROGER, GMOGER, GAOGER, GLOGEN	

The binding properties of C2C12 α1+ and α10+ were very similar (Fig. 2) except for on Col III where C2C12-α1+ cells showed greater attachment than α10+ cells. This may suggest that either Col III contains auxiliary motifs alongside GLOGEN, which possess different affinities towards integrins α1 and α10, or alternatively that there are differences in the display of GLOGEN sequences on Col III towards α1 and α10 receptors.

The similar adhesion levels observed on short synthetic peptides and their longer Toolkit equivalents seem in contradiction with the predicted density of cell recognition sequences due to the different masses of these peptides. Each peptide contained a single active collagen-recognition motif (GFOFER or GLOGEN); however the shorter peptides contain 42 amino acids (AA) whilst the larger Toolkit counterparts are considerably longer (63AA). As a consistent mass of peptide was applied to the tissue culture plate surface, this implies that the active ligand density of the short peptide might be 50% higher that of the longer Toolkit peptide, assuming uniform monolayer coating. However, this apparent difference in density does not invoke a corresponding increase in cell adhesion. This may be due to a number of reasons. Firstly, it is possible that cell binding is saturating on the longer peptide and so the higher density of the shorter peptide cannot elicit a further effect. Secondly, it could be that the larger, native-derived Toolkit peptides have a higher affinity for cell integrins in comparison to the shorter sequences due to differences in flexibility and in conformation of their triple-helical structures. There are differences in transition temperatures (T_m_) between short peptides and Toolkit peptides. The T_m_ of GFOGER is almost 10 °C higher than that of Toolkit peptides: Tm of GFOGER = 56 °C [35] and T_m_ of II-28 and III-7 were 46 °C and 45 °C, respectively (data not published). It is possible that this reflects a tighter, more compact triple-helical configuration of GFOGER than the longer Toolkit peptides. Native collagen approximates to 10-fold axial symmetry, whereas the GPP-containing flanking sequences adopt 7-fold symmetry. The transition between the two is not abrupt [13], [15], so that the helical twist of the GFOGER motif differs slightly depending on its local setting, and may influence the position and exposure of its amino acid residues (especially E) to integrins. It is also possible that the longer Toolkit peptides retain their native-like triple-helical conformation upon surface immobilisation resulting in greater activity towards cell receptors. Finally, the Toolkit peptides include primary sequence on each side of the active motif which may contribute to better recognition by native integrins. In accord with this theory, in the case of Rugli cells, with rat integrin α1, lower adhesion levels on shorter in comparison to the corresponding longer Toolkit peptides was noted. It is possible that the rat integrin α1 is more sensitive to the collagen conformation, indicating that a native-like structure of adhesion motifs is critical for their activity. This is consistent with numerous reports showing that cell adhesion to GxOGEx’ motifs is dependent upon a triple-helical conformation. This theory may explain, to some extent, the similar level of adhesion found on complete collagen molecules compared with the corresponding peptides which, due to their differential mass, would possess different surface densities (almost 15–25 times higher on peptides than on full collagen molecules). It is also likely that adsorption efficiency to the well surface of large collagen molecules differs from the comparatively short synthetic and Toolkit peptides which may affect the exposure of their cell-adhesive motifs. To finish, it is plausible that changes in the topology between the intact collagen molecules and peptides coatings, for example large fibrils on fibrillar collagens *vs* small structures on both types of peptides, may result in altered integrin ligation.

Cellular spreading analysis was performed to determine whether cation-dependent integrin-mediated cell adhesion could elicit flattened native-like cell morphology. Spreading of HT1080, Rugli and C2C12-α2+ cells, all expressing collagen-binding receptors, was high on all Col-based surfaces. This indicates that these substrates are capable of stimulating cell-signalling mechanisms that lead to spreading. As such it appears that integrin-mediated cell engagement with collagen can induce cell spreading via both integrin α2 and α1. Moreover this is not sensitive to the human or rat cell origin, showing that they interact with collagen substrates in a physiologically relevant manner leading to a very similar level of spreading that is independent on the collagen type and tissue source.

### 4.2 Adhesion on films

Adhesion tests were carried out on 2-dimensional films obtained from insoluble tendon and skin derived Col I and cross-linked with different doses of EDC. As Col I continue to be a predominant choice for the design of protein-based biomaterials the understanding of its adhesive characteristics, when derived from different tissues, and analysed with a diversity of cellular models, as presented here, may assist in the selection of optimum structural components for collagen-based cell supports.

EDC crosslinking is one of the most effective treatments for improved structural stability, resistance to dissolution and tailored mechanics of collagenous matrices with implications for their clinical use as tissue engineering biomaterials. As EDC-mediated bonding impacts on cell-substrate interactive properties it is important to balance the need for structural integrity and mechanics against bioactivity in each specific clinical case. In our previous works we investigated the effect of reducing the EDC concentration down to very low levels (up to 100 times dilution, 1% EDC, with respect to standard condition) on the physical and cell-interactive properties. We demonstrated, for example, that the EDC/NHS crosslinking dose influences both the level and the mode of cellular engagement to tendon-derived Col [32]. Cell attachment changed from predominantly integrin-mediated in the absence of EDC treatment, to divalent cation-independent with high EDC crosslinking concentrations (between 100 and 500%). In this work, we further assess the influence of EDC crosslinking on the ligation of cells with single, defined, integrin populations to collagen substrates. Therefore, we were interested in the effect that the collagen source induces on cell adhesion and, additionally, if cell adhesion sites on dermal and tendon-derived samples were equally altered by carbodiimide-mediated bonding. In contrast with the earlier study [32], here, through the use of specific experimental conditions, we focused on only physiologically relevant cation-dependent integrin-mediated anchorage of the four different collagen-binding integrins to collagen substrates. In particular, we introduced modifications to the cell adhesion methodology compared with that employed in the cited work [32]. This involved limiting the crosslinking concentration to 200% EDC (maintaining consistent the dilution levels up to 1%) and conducting the cell adhesion assay on the bench, at room temperature and without CO_2_. In addition, a more stringent washing regime was used to ensure the complete removal of loosely-bound or non-Mg^2+^-dependently attached cells from material surfaces. Through these modifications the non-cation dependent adhesion was limited, allowing us to examine only intimate, strong cell-substrate interactions. As such, this minimised lower affinity non-native-like collagen binding. Using this experimental approach, our results showed that crosslinking strongly decreases integrin-mediated cell binding of all cell lines via all four collagen-binding integrins (α1β1,α2β1, α10β1 and α11β1) to all films. The shape of the adhesion curves (A 490 nm vs EDC %) for HT1080 and C2C12 transfected cells resemble that found for the amine group content on Col (S) and Col (D) as a function of EDC concentration [24]. According to the well-established crosslinking mechanism, the number of amine groups involved in the EDC-promoted bonding is proportional to the quantity of carboxylic moieties on the AA residues (such as glutamate and/or aspartate) participating in the linkage. These same carboxylic groups are critical for cation-coordination between I domains of integrins and collagen and so EDC crosslinking, by consuming important chemical elements of the cell recognition sequences in collagen, depletes cell attachment to highly crosslinked collagen. These results are in agreement with our previous reports [18], [24], [29] where we hypothesise that carbodiimide treatment of collagenous materials significantly decreased the content of important carboxylic groups on glutamate and aspartate amino acid residues.

The adhesion of transfected C2C12 cells on films showed that the collagen origin substantially influenced the adhesion profiles. Comparison between the transfected lines showed that this adhesive profile was sensitive to the class of integrin involved in cell-substrate ligation. Contrary to the ligation levels observed on coatings, the adhesion values for almost all receptors, with the exception of α2, were higher on dermal collagen films than on their tendon-derived equivalents. This lower adhesion observed on dermal collagen monolayers could be attributed to a number of factors including the surface protein density, differences in collagen preparation from each source, or possible conformational changes due to surface interaction. Our films were ∼8 µm thick, meaning that the surface cell-recognition ligands are separated from the underlying plastic by a large number of collagen molecules. As such, it is highly unlikely that any surface-induced alteration of the collagen structure would be propagated to the surface and so influence the binding of cells to Col films. By contrast, on monolayers, the plastic could influence the structure of collagen molecules exposed to cells. Interestingly this higher adhesive level on Col I(D) films was noted for α1+, α10+ and α11+ but not α2+ C2C12 cells. As integrin α2 has a different affinity for cell-binding motifs in collagen, this may point to differences in the number or display of certain triple-helical motifs on dermal versus tendon collagen. It is also possible that bulk material properties may modulate, to some extent, integrin engagement to the films but not the monolayers. For example, we have found that the resistance to compression of matrices obtained from tendon collagen is higher than that of dermal (unpublished results). Therefore the cells may be responding differently to the mechanical environment in conjunction with the specific integrin binding sequences involved. This is in accordance with reports suggesting that integrins play a significant role in propagating mechanical cues to cells even at an early stage of cell-substrate interaction [1].

Differences were noted in the adhesion profiles of cells transfected with different integrins as a function of EDC-dose. This may point to differential sensitivity to EDC crosslinking of each of the glutamate-containing GxOGEx’ cell binding motifs. Each motif has a different integrin binding affinity, and so an unequal alteration of these cell recognition motifs by EDC may, in turn, alter the specific integrin binding profile of collagen I. EDC crosslinking up to 10% EDC concentration did not lead to an appreciable decrease in the adhesion of α2+ and α11+ positive C2C12 cells. These lines bind predominantly to high affinity GFOGER motifs, suggesting that the amount of unmodified GFOGER in low crosslinked samples was still sufficient to maintain cell adhesion, despite consumption of some glutamate (E) residues. Conversely, the EDC-induced decrease in α1 and α10 mediated cell binding to other GxOGER sequences, for example GLOGER, seems to be more sensitive to crosslinking. This may explain the observed drop in adhesion values for α1+ and α10+ cells even at low, 3% EDC concentrations. This effect is even more evident in Rugli cells with rat integrin α1 than for human integrin α1 containing C2C12 cells suggesting species-dependent affinity modulation of this receptor.

### 4.3 Attachment of -α1 human I-domain and -α1-rat I domain

Integrins α1β1 and α2β1 bind to collagen via inserted I-domains within the α-subunit (Fig. 1, [7]). Therefore, isolated recombinant human and rat integrin α1 I domains were used to assess integrin-collagen engagement in a cell-free system. The results agreed with the differences found in the adhesion of cells expressing human and rat integrin α1β1. They show that the difference in C2C12-α1+ and Rugli cell adhesion (with human and rat α1 receptors, respectively) in response to the dose of EDC used for collagen crosslinking was due to differential I domain binding. The observed lack of rat integrin α1 I domain engagement with GFOGER and with II-28 peptides (Fig. 8A and B) is consistent with low adhesion of Rugli cells on Col I/II coatings and these peptides. By contrast, C2C12 cells transfected with human α1 showed high adhesion levels on the same coatings which agree with the high affinity of human integrin α1 I-domain to GFOGER collagen sequences. In general, we have showed that while human integrin α1 I domain ligates to a wide variety of collagen derived triple-helical ligands, rat I domain shows high specificity almost exclusively to GLOGEN-containing sequences, that is synthetic GLOGEN and its Toolkit equivalent III-7 (Fig. 7A). These motifs are not present in Col I (see Table 2) and so rat integrin α1 binding to Col I is dependent upon lower affinity motifs. Therefore even a small decrease in the availability of these lower affinity sites, for example through EDC crosslinking, may lead to the significant loss of collagen binding capacity of cells expressing rat integrin α1. At the same time, cells which contain the human α1 I domain bind to a wider variety of similar triple-helical sequences including the GFOGER motif in Col I (Fig. 8A) and were much less sensitive to crosslinking. Presumably this is due to the higher affinity of GFOGER for human but not rat integrin α1. Additionally the different ligation behaviour of human and rat integrin α1 I domains may also explain the considerably elevated adhesion of cells with human α1 on Col I and II as this can occur through high affinity GFOGER motifs. Together these results show that both the integrin class and species-specificity dictate the affinity of these receptors to cell recognition motifs in different members of collagen family.

## 5 Conclusions

Integrins play a vital role in determining the cell response to environmental cues during the early stage of cell-substrate interaction. Therefore a detailed understanding of integrin binding to collagens, as widely used in biomaterials fabrication, is pivotal for endowing these materials with appropriate bioactivity. Here, we found that the cellular response to both the collagen type and origin is highly dependent on the specific integrin type and species being studied. This is due to integrin I domain interactions with collagen as shown using recombinant human and rat integrin α1-I domain binding assays. As for whole cell assays, these exhibited receptor class and species specificities of collagen interactions. Combining the results of cell attachment on differently crosslinked collagen films with those obtained in integrin α1-I-domain bindings studies, the biological response of human and rat cells (with the same α 1 integrin) to changes in the collagen type and origin and the EDC crosslinking status were elucidated. We have shown that EDC treatment decreases the overall amount of cell recognition sequences, whilst simultaneously altering the adhesive ratio among the different motifs on collagen molecules which further affected the specific integrin class that ligates to these collagen substrates after crosslinking. This comprehensive study of all four of the collagen-binding integrins gives key guidance in selection of the correct cellular model for the biological testing of biomaterials. For example we clearly show that rat models, which are used extensively for biological studies, may give different results from human-based systems. In summary, our data show that the collagen precursor should be carefully chosen dependent on the cell type to be incorporated and on the tissue to be replaced. This will assist in the selection of optimal materials for the design of cellular supports for different TE applications.
