# Supplementary material for: Selecting the correct cellular model for assessing of the biological response of collagen-based biomaterials
Source: Acta Biomater. 2018 Jan;65:88–101. doi: 10.1016/j.actbio.2017.10.035 (PMC5729022; doi:10.1016/j.actbio.2017.10.035)
Supplement: Supplementary data [file mmc1.docx]

**Detection of Collagen in coatings**

ELISA detection of collagen was used to verify surface coatings produced from insoluble suspensions and soluble solutions of collagen I. A mouse monoclonal anti-collagen I primary antibody (clone COL-1, Sigma, C2456) and a goat polyclonal anti-mouse HPR secondary antibody (DAKO) were used for this detection of collagen I. Insoluble Col I (Sigma) and (Devro) and soluble pepsin digested Col I (Devro, from bovine skin) were coated on the surface of Immulon 2HB 96-well plates (Thermo Scientific) as described in 2.2. Non-coated wells were employed as a negative control. Prior to detection, wells were blocked with 3% (w/v) BSA in binding buffer (TRIS buffered saline (TBS - 50mM TRIS, 140mM NaCl, pH 7.4) containing 1mg/mL BSA) for 1 h at room temperature. Following BSA blocking, the samples were washed with 3x200μL of binding buffer and then incubated for 1 hour at room temperature with 100μL of the primary antibody at a dilution of 1:1000 in TBS. The samples were washed in 3x200μL of binding buffer and subsequently incubated with 100μL of 1:10,000 diluted secondary antibody for 1h at room temperature. The wells were washed with 3x200μL of binding buffer then 100μL of TMB substrate (Thermo Scientific) was added to each well and the reaction was stopped after colour had developed by the addition of 100μL of 2.5M H_2_SO_4_ to each well. The absorbance at 450 nm, A_450_, was measured using a Spectra Max 190 (Molecular Devices). Values represent means of six measurements ± standard deviation. Statistical significance was calculated using a student t-test.

**Result**

The results of the collagen detection assay are presented in Figure 1.


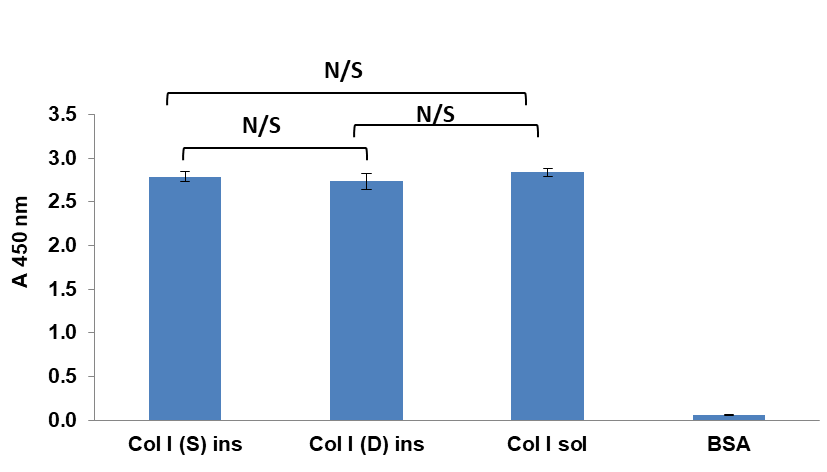


Fig.1 Detection of collagen on the different collagen coatings

These results show identical Col I detection on the surfaces of wells coated with insoluble Col I (S) and Col I (D), and soluble Col I preparations. This suggests that there is no significant difference between the adsorption of insoluble and soluble collagens and between the skin and tendon derived insoluble collagens. The standard deviation is very small and comparable between coatings produced from suspensions of insoluble samples and a solution of soluble collagens. Therefore the selected coating parameters used here, such as homogenizing conditions, low concentration of collagen suspensions and the amount of substrate per well, produce comparable coatings of both soluble and insoluble collagens. As such, these coatings represent suitable substrates to analyse the cell binding properties of these collagen sources.
